# Aircraft Noise Effects on Sleep—Results of a Pilot Study Near Philadelphia International Airport

**DOI:** 10.3390/ijerph16173178

**Published:** 2019-08-31

**Authors:** Mathias Basner, Maryam Witte, Sarah McGuire

**Affiliations:** Unit for Experimental Psychiatry, Division of Sleep and Chronobiology, Department of Psychiatry, University of Pennsylvania Perelman School of Medicine, Philadelphia, PA 19104, USA

**Keywords:** noise, sleep, health, awakening, arousal, aircraft, airport

## Abstract

Current objective data on aircraft noise effects on sleep are needed in the US to inform policy. In this pilot field study, heart rate and body movements were continuously measured during sleep of residents living in the vicinity of Philadelphia International Airport (PHL) and in a control region without aircraft noise with sociodemographic characteristics similar to the exposed region (*N* = 40 subjects each). The primary objective was to establish the feasibility of unattended field measurements. A secondary objective was to compare objective and subjective measures of sleep and health between control and aircraft noise exposed groups. For all measurements, there was less than 10% of data loss, demonstrating the feasibility of unattended home measurements. Based on 2375 recorded aircraft noise events, we found a significant (unadjusted *p* = 0.0136) exposure-response function between the maximum sound pressure level of aircraft noise events and awakening probability inferred from heart rate increases and body movements, which was similar to previous studies. Those living near the airport reported poorer sleep quality and poorer health than the control group in general, but when asked in the morning about their last night’s sleep, no significant difference was found between groups. Neither systolic nor diastolic morning blood pressures differed between study regions. While this study demonstrates the feasibility of unattended field study measurements, for a national study around multiple US airports refinements of the study design are necessary to further lower methodological expense and increase participation rates.

## 1. Introduction

According to the US Bureau of Transportation Statistics, approximately 9.4 million Americans (2.88%) were exposed to average 24-h aircraft noise levels exceeding 50 dB in 2017 [1]. The most recent US sleep studies on the effects of aircraft noise on sleep date back to 1996 [2]. Since then, US air traffic has changed significantly with changes in traffic volume and significant reductions in noise levels of single aircraft [3]. Due to differences, e.g., in building structure, the use of central and window air conditioning, airport operational procedures, and sleep timing, results from studies performed outside the US may not transfer directly to US domestic airports. Therefore, it is important that field studies be conducted in the US to acquire current data on sleep disturbance relative to varying degrees of noise exposure.

The gold standard for measuring sleep is polysomnography, which is the simultaneous measurement of brain electrical potentials (electroencephalogram, EEG), eye movements (electrooculogram, EOG), muscle tone (electromyogram, EMG), and other signals (e.g., respiratory movements, airflow, leg movements) to diagnose sleep disorders. Sleep stages are identified based on specific patterns in the physiological signals for each 30-s segment of the night [4]. Wake time is differentiated from sleep, and Rapid Eye Movement (REM) sleep is differentiated from non-REM sleep (stages S1 through S4). Stages S1 and S2 (N1 and N2 in the newer AASM criteria [5]) are considered light sleep and S3 and S4 (N3 in the AASM criteria) are considered deep sleep. Shorter activations in the EEG and EMG of 3 s or longer can also be scored and are referred to as cortical arousals.

Polysomnography has been implemented in a few field studies on the effects of road, rail, or aircraft noise on sleep [6,7,8,9]. However, it is expensive to implement as trained staff are needed to apply and remove the electrodes. Trained staff are also needed to visually score sleep stages, which has both high intra- and inter-rater variability [10,11]. Also, the methodology is somewhat invasive and may influence sleep itself, especially during the first night(s) [12]. A less invasive method for monitoring sleep is actigraphy, which infers sleep and wake patterns from body movements, measured using a wrist-worn device. While this approach is noninvasive and less expensive, analysis is typically based on 60-s segments and different algorithms are used to score the data. Compared to polysomnography, actigraphy has high sensitivity in identifying sleep epochs but a low specificity in identifying wake epochs [13,14,15].

Awakenings are typically associated with arousals of the autonomic nervous system, which include increases in heart rate and blood pressure. Basner et al. previously developed an algorithm for automatically identifying cortical arousals of 3 s or longer in duration based on increases in heart rate alone [16]. As these brief arousals can occur over 80 times a night without noise exposure, they are not considered a specific indicator of noise-induced sleep disturbance [17,18]. Therefore, during an earlier stage of this project, this algorithm was refined in order to only identify cortical arousals that are 15 s or longer in duration [19], which is the indicator of noise-induced sleep disturbance most commonly used in the field and a more specific indicator of sleep disruption [18]. Body movements measured with actigraphy were also newly included in the algorithm. The agreement between cortical arousals identified visually based on polysomnography data and arousals identified using the refined ECG- and actigraphy-based algorithm was evaluated by calculating Cohen’s Kappa, which represents agreement corrected for chance. A Kappa value greater than 0.80 was found, which is considered as a “near perfect” agreement between the two approaches according to conventional standards [20]. An advantage of using ECG and actigraphy only for monitoring sleep is that participants can apply the equipment themselves; therefore, reducing the methodological study cost as staff are not needed in the field each night and morning. In addition, the combined ECG/actigraphy device used in this study only required 2 chest electrodes (1 derivation of the ECG) compared to the multiple electrodes and wires that are required for polysomnographic sleep studies, which may have an effect on an individual’s sleep quality. Finally, the algorithm that was developed allows arousals to be identified automatically and consistently across studies.

The methodology of using ECG and actigraphy to monitor sleep was implemented in a pilot study that was conducted around Philadelphia International Airport (PHL). Eighty participants were enrolled in the study, with each participant completing three nights of unattended sleep measurements. Forty participants were recruited from regions near PHL airport and 40 were recruited from regions without relevant air-traffic in Philadelphia County. The primary objective of this study was to evaluate the feasibility of the study methodology, in particular the quantity and quality of data that could be obtained when participants use physiological and noise measurement equipment unattended. A secondary objective of this study was to compare objective and subjective measures of sleep and health between control and aircraft noise exposed groups.

## 2. Materials and Methods

### 2.1. Airport Selection and Noise Modeling

**Figure 1:**
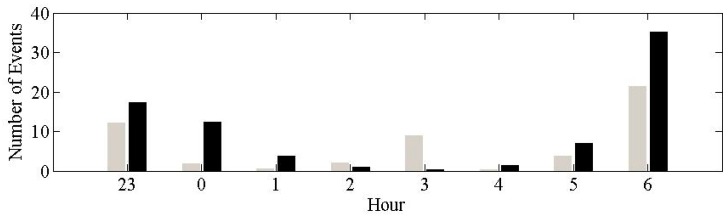
Average number of hourly operations at Philadelphia International Airport (PHL) based on 68 nights of modeled data (period June 2012 to September 2012). Departures (gray) and arrivals (black).

For this study, staff needed to go into the field to deploy and collect equipment. Therefore airports within proximity of the University of Pennsylvania were considered. In addition, the airport had to have relevant amounts of night-time air-traffic. Operations around PHL were examined in order to determine whether it met this requirement. PDARS (Performance Data Analysis and Reporting System) data was obtained from the Federal Aviation Administration (FAA) for PHL, and 4 months of data from June 2012 to September 2012 were modeled. The average number of hourly operations was calculated for 68 nights (Figure 1). There were 130 events between 11:00 p.m. and 7:00 a.m., with cargo operations in the middle of the night between 3:00 a.m. to 4:00 a.m.

**Figure 2:**
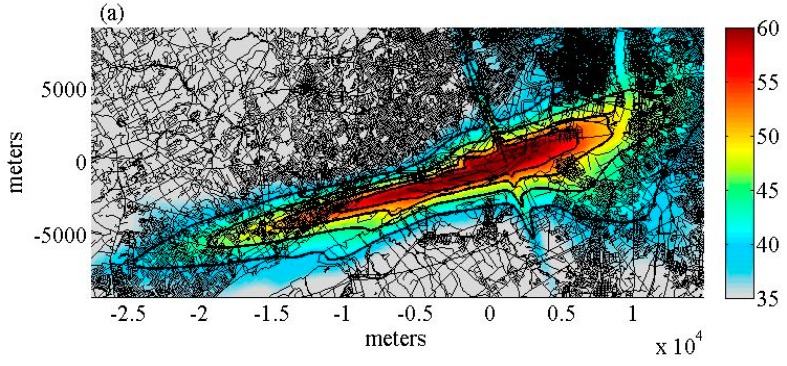

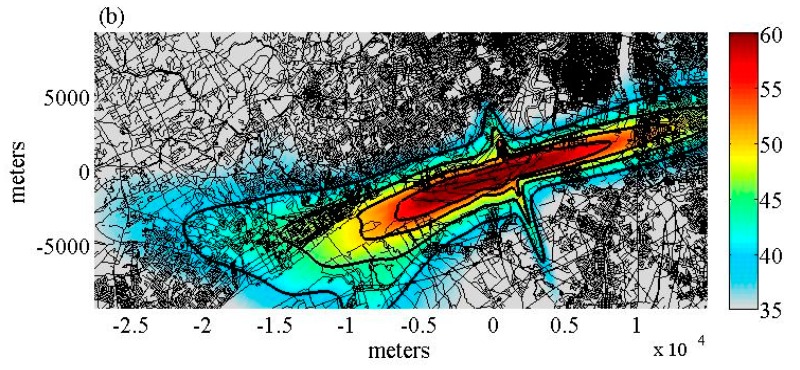
Average L_night_ contours for (**a**) East flow configuration (13.2% of modeled nights) and (**b**) West flow configuration (86.3% of modeled nights). The contour lines shown are the 40, 45, 50, and 55 dBA L_night_ contours.

For each of the 68 nights it was also determined whether the airport was operating predominantly in the East flow or West flow direction. For the East flow, the departures and arrivals are on runways 08, 09L, and 09R. For the West flow, the departures and arrivals are on runways 26, 27R, and 27L. The primary direction was in the East flow for 9 modeled nights and in the West flow for 59 modeled nights. Contours based on aircraft noise levels energetically averaged over the period 23:00 until 7:00 (L_night_) were calculated for each of the 68 nights using the Integrated Noise Model (INM), version 7.0d. The average L_night_ contour was calculated for the East flow and the West flow directions (Figure 2).

The total population within each contour was calculated using block level population data from the 2010 US Census, listed in Table 1. As PHL had a sufficient number of night-time flight operations and a large enough exposed population to sample from, it was selected as the site for the study. We recruited participants from Tinicum Township in Pennsylvania, situated West of the airport, and from Gloucester City, New Jersey, situated East of the airport. The distance between the center of the runway system of PHL airport and households participating in the field study was approximately 0.5–1.5 km (Figure 2). Both regions had comparable sociodemographic characteristics.

### 2.2. Control Region Selection

**Figure 3:**
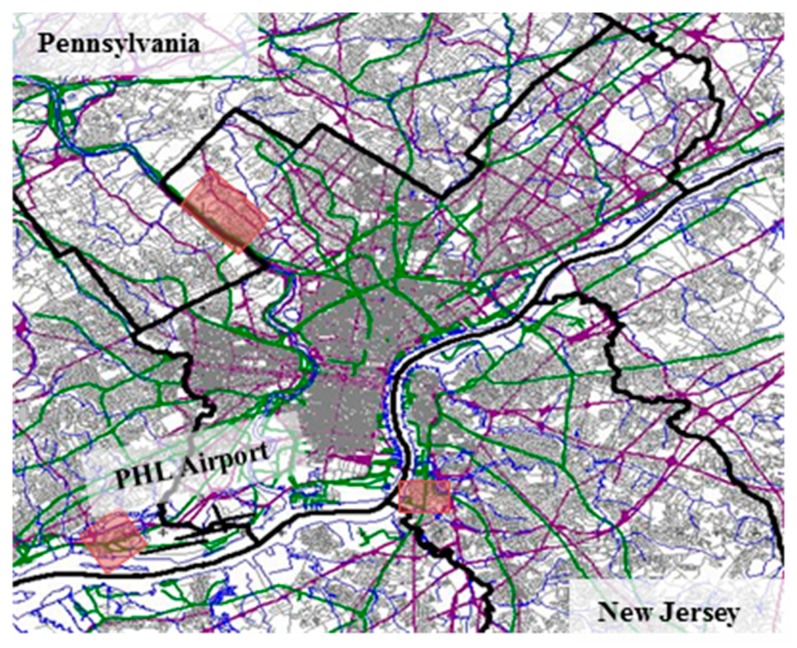
Measurement regions for the pilot sleep study conducted near Philadelphia International Airport. Measurement areas are highlighted in red.

The control region was selected based on socio-demographic and noise characteristics of the region in comparison to the aircraft noise exposed region around PHL. The sociodemographic data were extracted from the US Census 2012 American Community Survey. Data for Delaware, Philadelphia, and Montgomery County were obtained and plotted on the geographic level of the US census tract. We selected a region in Northwest Philadelphia County as the control region that had similar sociodemographic characteristics (median income $29,700–$73,600; >80% Caucasian) as the noise exposed region near PHL but was not exposed to relevant levels of aircraft noise (<5 predicted aircraft noise events with maximum sound pressure levels of >50 dBA during the night). All three sampling regions are shown in Figure 3.

### 2.3. Study Methodology

The protocol of the pilot study was approved by the Institutional Review Board of the University of Pennsylvania (Protocol #819770). The study for each participant lasted for 4 days/3 consecutive nights and either took place on Monday through Thursday or Tuesday through Friday, depending on the participant’s availability. Subjects were compensated with $50 for each night they participated. This study was restricted to weeknights only for consistency across the 3 nights for each subject, as bedtime, sleep duration, and flight schedules may be different on weekends. Two staff members went to the participant’s home on the first night of the study to explain the study protocol and walk participants through equipment use, obtain written informed consent, and setup equipment for monitoring the noise. It took approximately 1–2 h for each setup. The study measurements were then completed unattended for the next three nights, with staff members returning after the third night to collect the equipment, which required approximately 30 min. Staff members were available throughout the study via cell phone to address any questions or concerns that participants had.

#### 2.3.1. Physiological Measurements

During the night, participant’s sleep was monitored using one device (eMotion Faros 90) that measured both heart rate and body movements. The device was battery powered and attached with two electrodes to the chest of the subject. The ECG was sampled at 1 kHz and the peak of each R-wave was detected and recorded. Movement was also measured using a 3-axis accelerometer at a sample rate of 10 Hz, 14 bit resolution. As movement was recorded with a high resolution, breathing patterns could be inferred from movements of the chest and it could be determined whether participants had chest movements that would be suggestive of sleep apnea during the night.

To examine potential consequences of noise-induced sleep disturbance, participants completed blood pressure measurements each morning in an upright seated position using a home monitor with pre-formed arm cuff for 9–17 inches (Omron BP791IT). Three consecutive measurements were taken automatically with one min intervals between measurements. Participants were told not to drink caffeine, smoke, or exercise, and to be sitting in a state of rest for 5–10 min before completing the measurements.

#### 2.3.2. Environmental Measurements

To monitor the noise in the participant’s bedroom, one microphone was set up near the head of the bed. The height of the microphone was 1 foot above the mattress, i.e., approximately the height of the participant’s head on the pillow. Due to furniture in the room, it was not always possible to place the microphone directly next to the pillow, sometimes it had to be set up at the foot of the bed so that the participants could move around their bedroom comfortably. One second energetic A-weighted average noise levels (L_Aeq_) and unweighted one-third octave band levels were recorded 24 h a day throughout the study using a class-1 sound level meter (Larson and Davis Sound Level Meter 831). At night before going to bed, participants turned on an additional sound recorder (Roland R-05) which saved standard resolution audio recordings (16-bit, 44,100 Hz, .wav files) of the sounds inside the bedroom. Sound recordings during the night were made so that the source of noise events could be determined. If there were two participants in the same room, only one microphone and set of sound recording equipment was used. Participants were instructed to start and stop the sound recorder when the first person retired to bed and after the last person woke up in the morning, respectively.

A less expensive audio recorder (Tascam DR-07) was placed outside near the participant’s bedroom window. Standard resolution audio recording files were saved (16 bit, 44,100 Hz, .wav files). Sounds were recorded 24 h a day. The recorder was placed outside on a weighted tripod or placed on the window ledge. The purpose of the outdoor recordings was for identification of the noise source only; outdoor sound recorders were not calibrated.

#### 2.3.3. Subjective Assessments

Each morning participants completed a brief questionnaire on their previous night’s sleep that included questions on window position, time they went to bed and switched off the lights, time they woke up, time they got out of bed, duration it took them to fall asleep, sleep quality, noise during the night, and their level of fatigue in the morning. Subjects also completed four surveys on the first day of the study, three of which were on their sleep and health and included the Health Survey (SF-36) [21], the Pittsburgh Sleep Quality Index (PSQI) [22], and the Horne-Ostberg Morningness-Eveningness Questionnaire [23]. The participants also completed a questionnaire with sociodemographic questions. All questionnaires were implemented as web-based surveys using a system called Redcap, which is designed for collecting clinical research data. The surveys were completed using Apple iPads and automatically transmitted to the Redcap server via a cellular data network upon completion.

#### 2.3.4. Additional Protocol Instructions

Participants were allowed to go to sleep at their normal times and wake up at their normal times each night. Participants were asked to turn off any noise producing items such as the TV, radio, or music during the night. However, in order to preserve a regular or normal sleeping environment, participants were allowed to turn on fans, air conditioners and heaters for their comfort. Also, participants were allowed to sleep with their pets (such as dogs and cats) as they would have normally in their bedrooms. It was desired to have participants maintain as close to their normal sleep routine as possible.

#### 2.3.5. Subject Recruitment

Three methods were used to recruit participants for this study. The first approach was to go door-to-door. Staff members knocked on the door of every house on a block in the evening hours between 5:00 p.m. and 8:30 p.m. for a total of 35 blocks that had the required L_night_ levels. If household members were not home, a study flyer was left hanging on the door. Flyers on the study were also placed throughout the community on public bulletin boards at locations including the post-office, library, and community centers. A total of ten participants near the airport were recruited using these two approaches.

Due to the low response, the remaining 70 participants were recruited by mailing flyers to residences. All addresses within eight census tracts were purchased from a commercial vendor. For the control region, addresses were randomly selected from the list of addresses that were obtained. For the communities near the airport, the residents with the highest predicted night-time noise levels were selected. While the target enrollment of 80 participants was met using this approach, the response rate was still low with 3700 flyers mailed to obtain this enrollment.

Individuals interested in taking part in the study were screened over the phone to determine their eligibility. As few selection criteria as possible were used in order to increase response rates and the generalizability of results. Participants had to be 21 years or older. They could not be morbidly obese (Body Mass Index [BMI] over 35), as the risk for sleep apnea increases with increasing BMI. Also, participants could not have a history of cardiac arrhythmia or history of a sleep disorder (including obstructive or central sleep apnea, narcolepsy, restless legs syndrome or periodic limb movement syndrome). Furthermore, participants had to have normal hearing, not consume sleep medication on a regular basis, not work night shifts, or have children under five years old living in the same household. Interested individuals that were pregnant were ineligible. More than one person per household could take part in the study.

When relying on self-report for determining eligibility, there is the potential that participants have an undiagnosed condition. The heart rate and actigraphy data were examined after the 3 nights of the study to determine if the participants had either a cardiac arrhythmia or a sleep-disorder. If a condition was identified, the individual’s data was removed from analysis and a letter was sent to the participant recommending they see their medical doctor for further evaluation. In this study, only 2 participants were identified to potentially suffer from a sleep-related breathing disorder, and one from cardiac arrhythmia.

### 2.4. Data Analysis

#### 2.4.1. Acoustic Analysis—Aircraft Event Scoring

PDARS flight operations data was obtained for the time period of the study. A Matlab (Matlab version 2014b, Mathworks, Natick, MA, USA) program was written in order to identify aircraft events within the night-time noise recordings. The program calculates the distance between each aircraft’s flight path and the geocoded addresses of the participants. The minimum distance between the two is determined and an aircraft event is detected in the file at the time of the minimum detected distance. All events identified by the program were also verified by a human scorer. Each sound was listened to and systematically labeled. The 2 min preceding and following each aircraft event were also scored.

If additional sounds occurred at the same time as an aircraft event (e.g., outdoor events such as a car or train, indoor events such as snoring or turning over in bed), these events were also scored. Outdoor sound recordings were used if sounds were unidentifiable using the indoor sound recordings. For 46% of participants’ homes near the airport, periods of sounds were masked by high background noise levels due to heaters or air-conditioners and fans.

#### 2.4.2. Automatic Identification of Awakenings Based on Heart Rate and Actigraphy Data

Awakenings during the night were identified automatically based on the heart rate and actigraphy data. The software was based on the algorithm of Basner et al. [16], which identified EEG arousals (≥3 s) based on heart rate alone. This algorithm was refined to identify EEG awakenings (≥15 s) using heart rate and actigraphy data, which is a more specific indicator of noise-induced sleep disturbance due to the lower frequency of occurrence on nights without noise exposure [18]. Awakenings are identified in the algorithm by using matrices of likelihood ratios that indicate whether the difference in the beat-to-beat heart rate compared to a 3 min median heart rate or the amount of movement is associated with an awakening [19].

Awakenings were calculated for every subject night. After the calculations were completed, artifacts in the heart rate signals were visually identified, and these periods were removed from analysis. During periods in which the heart rate signal was invalid (6% of nights had invalid periods), awakenings were identified based on actigraphically determined movement only and included in the analysis. The accuracy of detecting awakenings based on actigraphy alone is somewhat lower relative to heart rate alone or heart rate and actigraphy combined. However, the agreement with polysomnography was still found to be almost perfect (kappa = 0.81; compared to kappa = 0.87 for actigraphy and heart rate combined).

#### 2.4.3. Time Drift Correction

The Faros 90 devices (Bittium Corporation, Oulu, Finland) had a time drift of up to 10 s over the 4 days. This was determined based on time synchronizations of the devices before and after each set of measurements. In comparison, the Larsen and Davis 831 Sound Level Meter had a stable time and only drifted on average 1 s over the 4-day period. To correct for the time drift between the two devices, the difference in the onset of movement detected in the actigraphy signal and detected audibly in the sound recordings was determined for at least 3 time points per night. The time of the awakenings was then corrected linearly. This time drift correction was necessary for the single event awakening analysis.

#### 2.4.4. Single Event Awakening Analysis

All aircraft events were included in the single event analysis regardless of whether another noise source occurred at the same time, such as an aircraft event occurring at the same time as a car pass-by. In analyses performed for the World Health Organization (WHO) based on data from the German Aerospace Center’s (DLR) STRAIN study, it was found that for aircraft noise, exposure-response relationships did not vary relevantly when including all events or only events that did not co-occur with noise events from other sources [19]. A 50-s time window extending from −5 s until +45 s relative to the start of each aircraft noise event was screened for an awakening. A noise event was excluded from analysis if an awakening started before the start of this screening window and extended into or even beyond it. Five seconds before the start of the aircraft noise event were added to the screening window to account for any inaccuracies in synchronizing acoustical and physiological measurement equipment (see Section 2.4.3). The 50-s duration of the screening window was derived empirically from data collected at four different airports (PHL, ATL, Frankfurt [FRA], and Cologne-Bonn [CGN]), which maximized slope estimates for the maximum sound pressure level.

#### 2.4.5. Statistical Analysis

Statistical analysis was performed using SAS (version 9.3, SAS Institute, Carey, NC, USA). For the calculation of single event exposure-response relationships for the probability of an awakening, logistic mixed models with random subject intercept were calculated using Proc NLMIXED. The random intercept term accounts for the correlation of the repeated observations within each subject. In this case the repeated observations are multiple reactions to aircraft noise events observed per subject. For all other outcomes, linear mixed effect models were calculated using Proc Mixed. A *p*-value of 0.05 or less was considered statistically significant.

We ran either models that included an indicator variable differentiating the airport region (airport = 1) from the control region (airport = 0), or models that included the average noise level as measured during the sleep period (L_Aeq_) as a continuous independent variable. Some models were adjusted for potential confounding. The variables age, BMI, and time from sleep onset were included as continuous variables, while sex was included as a nominal variable (value of 1 = male, 0 = female).

For blood pressure measurement analyses, the systolic and diastolic blood pressure levels were averaged across all 3 measurements for each morning. Mixed models with a random subject intercept compared study regions (airport vs. control) and were adjusted for age, sex, and BMI.

## 3. Results

### 3.1. Participant Characteristics

Eighty participants were enrolled in the study, and 79 completed the measurements. The participants were from 56 different households. The measurements were conducted over a one-year period between July 2014 and July 2015. Demographic characteristics for the 79 participants are listed in Table 2. While the mean age of participants near the airport was higher than for the control region, participants from both areas were of a wide age range. The majority of participants in both regions had at least some college education, and the percentage of participants who considered themselves noise sensitive was low in both areas. Noise sensitive was defined as reporting very or extremely sensitive to any kind of noise on the demographic questionnaire. For all remaining analyses, data for 3 participants (1 aircraft noise exposed and 2 control region) were removed due to potential health conditions.

### 3.2. Feasibility of Study Protocol

The primary objective of this study was to evaluate the quality of data that could be obtained by performing an unattended sleep study. Overall, it was found that participants were able to follow the study protocol well (Table 3). For 93.4% of the nights, there were no missing periods of ECG data due to participants not wearing the device or due to improper use of the device, electrodes, or cables. For 5.7% of the nights, partial ECG recordings were obtained and for only 0.9% of nights, no valid ECG data was recorded. For 93.4% of the mornings, participants completed all 3 blood pressure measurements and for 5.3% of the mornings at least one blood pressure reading was recorded. For 89.4% of the nights, full sound recordings were obtained. Data loss was due to either equipment problems or participants failing to turn on the sound recorder at night. All questionnaires for the study were completed. The surveys were web-based, which allowed staff members to verify completion of the surveys in real time and contact participants if the study protocol was not being followed. The compliance of participants in turning on the sound recorder, wearing the ECG device, and completing the morning blood pressure measurements each night/morning could not be tracked in real time.

### 3.3. Aircraft Noise Levels

**Figure 4:**
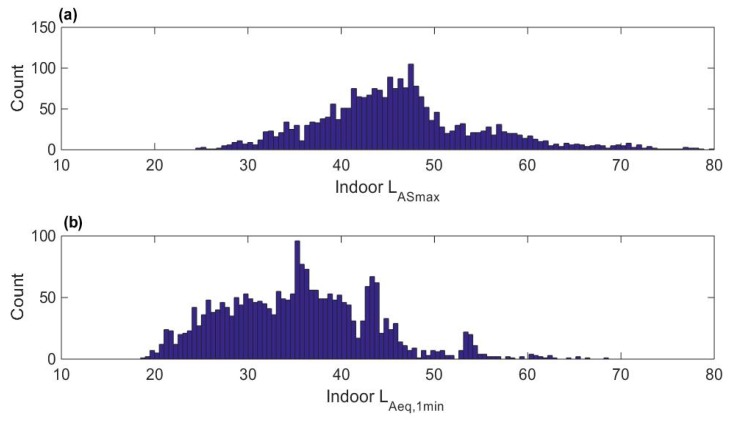
Indoor noise levels for participants near the airport. (**a**) L_ASmax_ of aircraft events; (**b**) L_Aeq_ 1 min before each aircraft event.

The distribution of indoor maximum noise levels for the aircraft events within participant’s homes near the airport is shown in Figure 4a. The total number of noise events within the sleep period for the participants near the airport was 2375. The median indoor maximum A-weighted sound pressure level with slow time constant L_ASmax_ of the aircraft events was 45.5 dBA. The average noise level 1 min preceding each event is shown in Figure 4b, and the median was 35.4 dBA.

**Figure 5:**
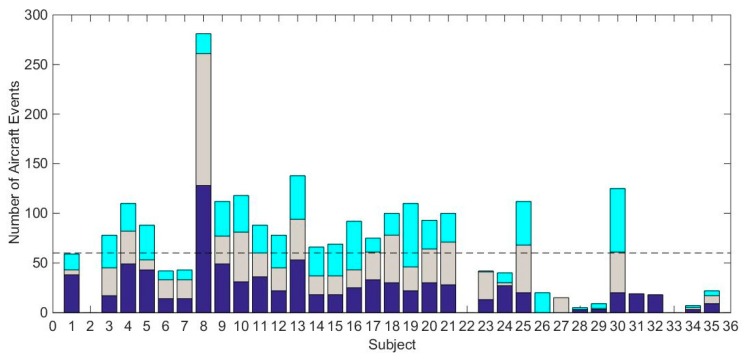
Number of aircraft noise events per subject near PHL airport for each night (4 out of the 36 participants had no audible events; this was due to masking noise from a TV, fan, or air conditioner). The colors indicate study nights 1 (purple), 2 (gray), and 3 (turquoise).

The number of events per night per subject who lived near the airport is shown in Figure 5. Out of the 38 participants in the aircraft noise group (1 subject consented but did not participate, and 1 subject was removed due to a potential health condition), noise measurements failed at 1 household which had 2 subjects. For the remaining 36 participants, the median number of aircraft noise events was 65.4. Four out of the 36 participants had no audible events; this was due to masking noise from a TV, fan, or air conditioner. Twenty of the 36 subjects had more than 60 events, which was the target when the study was designed.

The average indoor L_Aeq_ level for the sleep period time (SPT; the time between sleep onset and final awakening) was also calculated for each night and for individuals living near the airport and in the control region. The median L_Aeq_ level during the SPT for the noise exposed group and for the control group was 43.2 dBA and 31.8 dBA, respectively. While it was a goal of the study to have a control region completely without aircraft events, there were some overflights within the area. The median L_ASmax_ level of these events was 36 dBA.

### 3.4. Descriptive Sleep Parameters of the Entire Night

**Figure 6:**
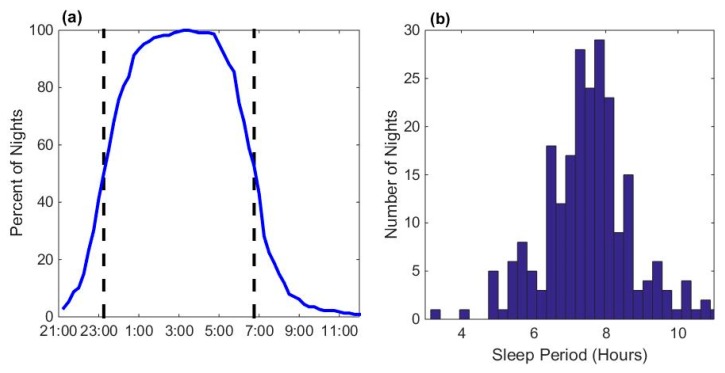
(**a**) Percentage of sleeping participants; (**b**) distribution of sleep period durations for all nights (*N* = 227 nights).

The cumulative distribution of the sleep period times calculated based on the heart rate and actigraphy data for all participants is shown in Figure 6. The majority of participants were asleep between 11:00 p.m. and 7:00 a.m. The median sleep period time was 7.5 h.

### 3.5. Effects of Aircraft Noise on the Sleep Fragmentation Index

The sleep fragmentation index was also calculated for each night. This index is defined as the number of awakenings divided by the sleep period time in hours. Mixed models with random subject intercepts were calculated and the results are shown in Table 4. Model 1 and Model 2 were adjusted for age, sex, and BMI. Model 1 investigated the effect of study region on the sleep fragmentation index. Model 2 contained the average noise level during the sleep period (L_Aeq_) instead of the region. In both models, the only variable that was significant was age, which was negatively associated with the sleep fragmentation index. We also added a term for age^2^ to determine whether there was a non-linear trend with age, but no significant effect was observed (*p* = 0.8037 in Model 1 and *p* = 0.8912 in Model 2 for age^2^).

### 3.6. Single Event Awakening Analysis

Random intercept logistic regression models were calculated for the probability of awakening to an aircraft. Model 1 contained only the indoor maximum noise level, Model 2 was adjusted for age, sex, BMI, and time from sleep onset (Table 5). A total of 2010 aircraft noise events contributed to the analysis. In both models, the coefficient for L_ASmax_ was positive and significant (i.e., awakening probability increased statistically significantly with increasing L_ASmax_). In Model 2, the probability of awakening was found to increase significantly with the time from sleep onset, consistent with previous findings [6,24]. Awakening probability also decreased significantly with increasing BMI.

**Figure 7:**
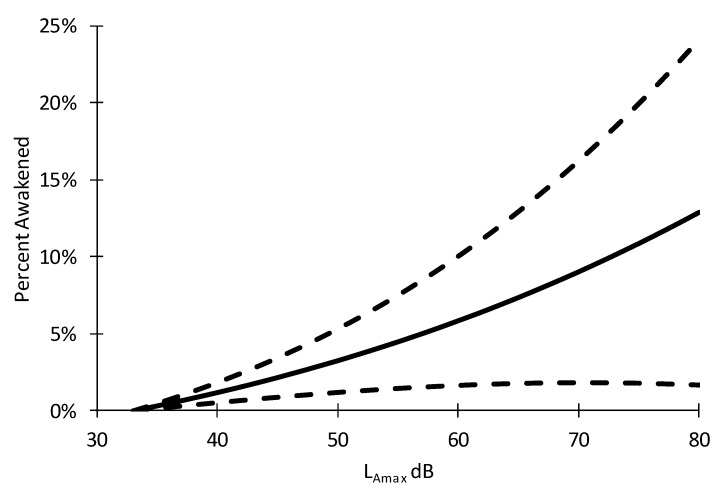
The unadjusted probability of an additional awakening induced by aircraft noise depending on indoor maximum sound pressure level L_Amax_ (slow time weighting) for PHL International Airport. Dashed lines indicate 95% confidence intervals.

The exposure-response relationship for additional awakenings due to aircraft events (P_noise_-P_spontaneous_) is shown in Figure 7 (this relationship is based on unadjusted Model 1 above). To account for spontaneous awakenings in the exposure-response function [25], an estimate statement was used in NLMIXED to subtract awakening probability at 33 dB from the awakening probability for maximum SPLs between 33 dB and 80 dB. The threshold of 33 dB was informed by (a) a previous field study on the effects of aircraft noise on sleep [6], and (b) by the median background noise level one minute prior to the start of the aircraft noise events in this study (35.2 dB). Listening experiments performed in our laboratory confirmed that humans are unlikely to perceive an aircraft noise event if its maximum SPL is more than 2–3 dB lower than a background white noise level. Due to the relatively low number of subjects and aircraft noise events per subject, the 95% confidence interval of the exposure-response function is relatively wide.

### 3.7. Blood Pressure Measurement Analysis

Systolic blood pressure increased significantly with age, BMI, and was significantly higher in male participants (Table 6). No statistically significant association was found for study region (airport/control) or the equivalent aircraft noise level L_Aeq_. For diastolic blood pressure, there was a statistically significant association with BMI and age, but not sex (Table 7). No statistically significant association was found for study region or the equivalent aircraft noise level L_Aeq_.

### 3.8. Results of Self-Reported Measures

#### 3.8.1. PROMIS Sleep Questions

The sociodemographic questionnaire that was filled out once on the first day of the study asked several questions about sleep that were based on the Patient-Reported Outcomes Measurement Information System (PROMIS) Sleep Questionnaire [26]. The participants were asked several questions about their sleep quality during the past month, the results are listed in Table 8. Each question had a 5-point response scale which ranged from never (1) to always (5). Linear mixed effect models adjusted for age, sex, BMI, and study region were calculated. The coefficients for the airport region are in Table 8. Several results were statistically significant with participants near the airport reporting their sleep as less refreshing (*p* = 0.0255), they had more difficulty falling asleep (*p* = 0.0267), and did not get enough sleep (*p* = 0.0235) compared to those living in the control region.

#### 3.8.2. SF-36 Health Survey

The SF-36 was filled out once on the first day of the study. The SF-36 survey contains several questions to evaluate an individual’s perceived health. Linear mixed models were calculated for several questions and adjusted for age, sex, and BMI. When participants were asked to rate their health from poor (1) to excellent (5), those living in the airport region tended to rate their health worse than those living in the control region, albeit statistically non-significantly (−0.4122, *p* = 0.0538). The coefficient for the airport region for several questions in which participants were asked to rate how true or false the statements were can be found in Table 9. Participants living near the airport rated that they expected their health to get worse (+0.60, *p* = 0.0308) and that their health was not excellent (−0.58, *p* = 0.0074) compared to the control region.

#### 3.8.3. Pittsburgh Sleep Quality Index (PSQI)

The PSQI was filled out once on the first day of the study. It retrospectively assesses sleep quality over a period of a month. Responses to individual questions on the PSQI survey were combined to obtain a global score, which ranges from 0 (indicating best sleep quality) to 21 (indicating worst sleep quality). Scores > 5 are typically used to distinguish poor quality sleep from high quality sleep. Linear mixed models adjusted for age, sex, and BMI were calculated for the global score (Table 10). Those living near the airport (mean PSQI 6.2, SD 2.9) had a significantly (*p* = 0.0180) higher global PSQI score, indicating worse sleep quality compared to the control region (mean PSQI 4.4, SD 1.8). In the airport region, 60.5% reported a PSQI score > 5 compared to 18.4% in the control region (*p* = 0.0061). Higher BMI was also significantly related to worse subjective sleep quality (*p* = 0.0420), while age and sex showed no statistically significant relationship (*p* > 0.05).

#### 3.8.4. Morning Survey

This survey was completed on every study morning (i.e., 3 measurements per subject) and contained questions on sleep quality and fatigue. Linear mixed models, adjusted for age, sex, and BMI were calculated to determine whether there was a difference in evaluations between the control and airport study region. Participants near the airport rated they were more tired (coefficient estimate for airport region: 0.4598). However, this did not differ significantly between the airport and the control region (*p* = 0.3481). There was also no statistically significant difference between regions for ratings of difficulty falling asleep (*p* = 0.9724) and sleep quality (*p* = 0.3231). Furthermore, no association was found between average noise levels during the sleep period and difficulty falling asleep (*p* = 0.7146) or sleep quality (*p* = 0.4517).

## 4. Discussion

The primary objective of this pilot field study was to evaluate the feasibility, and more specifically, the quantity and quality of the data that could be obtained when sleep and noise measurements were completed unattended. For all measurements, there was less than 10% data loss. Participants were able to correctly apply the electrodes and use the heart rate/actigraphy device. The primary reason for data loss was cables coming off the electrodes. However, actigraphy data was obtained in all cases. Additionally, participants turned on the sound recorder for the majority of nights. Overall, this demonstrates the feasibility of unattended physiological and noise measurements.

The second objective of this study was to evaluate whether there were differences in objective and subjective sleep and health measures between the airport and the control region. The sleep fragmentation index was higher in residents living near PHL airport relative to residents living in the control region, albeit statistically non-significantly. This can likely be attributed to the low statistical power of this pilot field study. It is somewhat surprising, though, especially since a significant exposure-response relationship between aircraft noise L_ASmax_ and awakenings inferred from body movements and ECG arousals was found. It is possible that airport residents were able to compensate for noise-induced awakenings during noise-free intervals [24]. Furthermore, the ECG-based algorithm is somewhat less sensitive in older subjects, and even though we adjusted for age in our models, residual confounding may have masked a higher sleep fragmentation in airport residents.

High blood pressure and cardiovascular disease have been shown to be associated with chronic exposure to aircraft noise [27,28]. However, in this study, we did not find a significant difference in either systolic or diastolic blood pressure between those living near the airport and those living in the control region. However, the power of this pilot study was likely too low to detect small differences in morning blood pressure.

For subjective responses, it was found that those living near the airport reported poorer sleep quality reflected in responses to the PROMIS and PSQI sleep questions, and poorer health as reported in the SF-36. It is currently unclear whether additional confounding variables that were not collected in the current study may account for some of these differences. The extension of this pilot study conducted around a different US airport collected more extensive information on noise exposure, attitudes, and health outcomes, and will thus likely shed more light on this question. The PROMIS and PSQI sleep questions referred to a one-month time frame. When participants were asked in the morning about their last night’s sleep, no significant difference was found between the airport and the control group.

An exposure-response model relating the indoor noise level of the aircraft events to the probability of awakening inferred from body movements and ECG arousals was also derived. Awakening probability increased statistically significantly with L_ASmax_ of aircraft noise events both in the unadjusted model and in the model adjusted for age, sex, BMI, and elapsed sleep time. The number of aircraft events of high noise levels was low in this study, as shown by the skewed distribution of noise levels. In addition, the total number of aircraft events contributing to this analysis was only approximately 2000. These two limitations led to a wide confidence interval for the estimated awakening probability. 

The long-term goal of this line of research is to derive exposure-response relationships that are representative for the US population exposed to nocturnal aircraft noise. This study was the first step in evaluating the feasibility of a study methodology for collecting unattended physiological and noise data to develop these models. Based on experiences in this study, further refinements of the protocol are needed. The target enrollment of 80 participants for the study was met, however, to recruit the participants, 3700 flyers were mailed. This low response rate limits the generalizability of the results. One contributing factor to the low response rate may be that, while the measurements took place unattended, staff members still had to enter participants’ homes to setup and collect the equipment. A website was created with information on the study which allowed individuals to verify both the study and study team. The link for the website was provided on the recruitment flyers. However, despite the website and the provided information, potential participants may still have been reluctant to allow unknown individuals into their home.

Another limitation of the study design was the methodological expense. This study required staff to be in the field from 2 to 4 days per week. If a multi-airport field study was conducted this way, trained staff would be required close to each of the measurement sites, which may not be feasible. In addition, the sound recording equipment used for this study cost several thousand dollars, which restricts the number of devices that can be purchased or available for use, restricts the number of sites that can be studied concurrently, and thus, also limits the sample size for the study. For this study, we had equipment to study three sites concurrently, which meant a minimum of 27 weeks of field work.

Visual identification of aircraft noise and other events was cumbersome and also requires trained staff. We made important progress in automatically identifying aircraft noise events based on sound level measurements and flight-track data. However, aircraft noise events were often masked by other indoor noise sources, especially air conditioning units. These masked aircraft noise events had to be excluded form data analysis. This needs to be taken into account for sample size analyses for future field studies in the US.

Finally, inferring awakenings from changes in heart rate and body movements has several advantages including the low methodological expense, the possibility of self-instrumentation, automatic analysis, and low invasiveness. However, the approach does not allow for a classification of sleep in stages, and therefore, an investigation of the effects of noise on sleep architecture. With the ongoing development and miniaturization of EEG technology, it may be possible to reliably measure the EEG with similar properties to the methodology used in our study in the future.

## 5. Conclusions

This study demonstrated the feasibility of obtaining high quality acoustic and physiological data in an unattended three-night field study on the effects of aircraft noise on sleep. However, the methodological expense was still high and participant response rates were low. In an extension of the pilot field study discussed here, we have thus modified the study design and finished data collection in the vicinity of another major US airport (data analysis is underway). Inexpensive yet reliable equipment for physiological and acoustical measurements was mailed out to participants, who then set up, used, and mailed back the equipment without the need of trained investigators on site. If this methodological approach proves feasible, it would allow high-quality yet cost effective measurements of large subject samples around multiple US airports, which are needed to inform future policies.

## Figures and Tables

**Table 1:** Number of residents within each L_night_ contour.

L_night_	Average East [*N* = 9]	Average West [*N* = 59]
>= 55 dBA	345	249
50–55 dBA	15,627	8901
45–50 dBA	39,183	41,596
40–45 dBA	278,672	83,011

**Table 2:** Socio-demographic characteristics of participants in the pilot study.

Characteristic	Aircraft Noise Exposed (*N* = 39)	Control Region (*N* = 40)	*p*-Value
Age (mean, range)	46, 22–77 years	32, 22–68 years	<0.0001 *
Male Sex	41%	48%	0.7243 ^†^
At least some college Education	67%	90%	0.0245 ^†^
Duration of Residence (mean)	11 years	6 years	0.0428 *
Noise Sensitive	13%	10%	0.9678 ^†^
Detached House	21%	13%	0.5113 ^†^
White Race	97%	90%	0.3708 ^†^

One subject in the aircraft noise exposed group consented but did not participate in the measurements. A *p*-value < 0.05 indicates values differ statistically significantly between the noise-exposed and control region. * Mann-Whitney-U Test; ^†^ Test of Equal Proportions with Yates’ continuity correction.

**Table 3:** Overview of data quality.

Nights of Study Completed (Total Subjects: 76)	
98.7% of subjects	Completed 3 nights/mornings
1.3% of subjects	Completed 2 of 3 nights/mornings
Heart Rate Measurements (Total Nights: 227)	
93.4% of nights	No missing recording periods due to improper use of device, electrodes, cables
5.7% of nights	Partial nights of ECG recordings
0.9% of nights	No valid ECG recording
Blood Pressure Measurements (Total mornings: 227)	
93.4% of mornings	3 of 3 blood pressure measurements completed
3.1% of mornings	2 of 3 blood pressure measurements completed
2.2% of mornings	1 of 3 blood pressure measurements completed
1.3% of mornings	0 of 3 blood pressure measurements completed
Indoor Sound Recordings (.wav files) (Total Nights: 227)	
89.4% of nights	Full recordings
7.9% of nights	Equipment problems
2.6% of nights	High background noise throughout night (e.g., TV)
Outdoor Sound Recordings (.wav files) (Total Nights: 227)	
94.7% of nights	Full recordings
2.6% of nights	No secure location to place device
2.6% of nights	Equipment problems
All questionnaires were completed.	

**Table 4:** Mixed model results for the sleep fragmentation index.

Variable	Model 1			Model 2		
	Estimate	SE	*p*-Value	Estimate	SE	*p*-Value
Age [years]	−0.0363	0.0160	0.0260	−0.0358	0.0160	0.0285
Male	0.5205	0.4234	0.2230	0.6816	0.4280	0.1160
BMI [kg/m^2^]	−0.0057	0.0537	0.9158	0.0153	0.0543	0.7791
Airport	0.1850	0.4760	0.6986			
L_Aeq_ [dB]				0.0036	0.0242	0.8809

SE: Standard Error.

**Table 5:** Random effect logistic regression models for the probability of awakening.

Variable	Model 1			Model 2		
	Estimate	SE	*p*-Value	Estimate	SE	*p*-Value
L_ASmax_ [dB]	0.0274	0.0092	0.0056	0.0262	0.0098	0.0117
Age [years]				−0.0092	0.0053	0.0936
Male				0.1817	0.1944	0.3574
BMI				−0.0513	0.0247	0.0464
Time [min]				0.0017	0.0005	0.0020

SE: Standard Error.

**Table 6:** Linear mixed effect regression models for aircraft noise effects on systolic blood pressure.

Variable	Model 1			Model 2		
	Estimate	SE	*p*-Value	Estimate	SE	*p*-Value
Age [years]	0.3903	0.0790	<0.0001	0.3514	0.0690	<0.0001
Male	10.3661	2.0976	<0.0001	10.0360	2.1050	<0.0001
BMI [kg/m^2^]	0.6647	0.2689	0.0159	0.6570	0.2695	0.0173
Airport	−2.3582	2.3773	0.3255			
L_Aeq_ [dB]				0.000021	0.00031	0.9460

SE: Standard Error.

**Table 7:** Linear mixed effect regression models for aircraft noise effects on diastolic blood pressure.

Variable	Model 1			Model 2		
	Estimate	SE	*p*-Value	Estimate	SE	*p*-Value
Age [years]	0.2245	0.0650	0.0009	0.2129	0.0564	0.0003
Male	2.9703	1.7253	0.0896	2.9760	1.7200	0.0878
BMI [kg/m^2^]	0.7517	0.2212	0.0011	0.7492	0.2202	0.0011
Airport	−0.7278	1.9552	0.7108			
L_Aeq_ [dB]				−0.000022	0.000245	0.9305

SE: Standard Error.

**Table 8:** Coefficient estimate for airport region based on linear mixed models adjusted for age, sex, and BMI for the listed sleep questions. Response categories were always (5), often (4), sometimes (3), rarely (2), and never (1).

Statement	Estimate	Standard Error	*p*-Value
My sleep was restless	0.2056	0.2163	0.3450
I was satisfied with my sleep	−0.3522	0.2279	0.1266
My sleep was refreshing	−0.4698	0.2059	0.0255
I had difficulty falling asleep	0.5771	0.2551	0.0267
I had trouble staying asleep	0.3472	0.2736	0.2086
I had trouble sleeping	0.3200	0.2300	0.1684
I got enough sleep	−0.4612	0.1991	0.0235

**Table 9:** Coefficient estimate for airport region for linear mixed models adjusted for age, sex, and BMI for the listed health questions. Response categories were (5) definitely true, (4) mostly true, (3) don’t know, (2) mostly false, and (1) definitely false.

Statement	Estimate	SE	*p*-Value
I seem to get sick a little easier than other people.	0.1548	0.2635	0.5586
I am as healthy as anybody I know.	−0.1939	0.2486	0.4380
I expect my health to get worse.	0.6035	0.2739	0.0308
My health is excellent.	−0.6145	0.2228	0.0074

SE: Standard Error.

**Table 10:** Coefficients for linear mixed models adjusted for age, sex, and BMI for the global Pittsburgh Sleep Quality Index (PSQI) score.

Variable	Estimate	Standard Error	*p*-Value
Age	0.0094	0.0211	0.6573
Male	−0.4071	0.5600	0.4697
BMI	0.1473	0.0711	0.0420
Airport	1.5227	0.6287	0.0180
